# Epidemiology, risk factors, and prediction score of carbapenem resistance among inpatients colonized or infected with 3rd generation cephalosporin resistant Enterobacterales

**DOI:** 10.1038/s41598-021-94295-1

**Published:** 2021-07-20

**Authors:** Rima Moghnieh, Dania Abdallah, Marwa Jadayel, Wael Zorkot, Hassan El Masri, Marie Joe Dib, Tasnim Omar, Loubna Sinno, Rawad Lakkis, Tamima Jisr

**Affiliations:** 1 Department of Internal Medicine, Division of Infectious Diseases, Makassed General Hospital, Beirut, Lebanon; 2 Pharmacy Department, Makassed General Hospital, Beirut, Lebanon; 3 Faculty of Pharmacy, Lebanese University, Beirut, Lebanon; 4 Faculty of Medicine, Lebanese University, Beirut, Lebanon; 5 Department of Internal Medicine, Makassed General Hospital, Beirut, Lebanon; 6 Department of Medical Research, Makassed General Hospital, Beirut, Lebanon; 7 Faculty of Arts and Sciences, American University of Beirut, Beirut, Lebanon; 8 Department of Laboratory Medicine, Makassed General Hospital, Beirut, Lebanon

**Keywords:** Diseases, Health care, Risk factors

## Abstract

In this study, we determined the incidence and risk factors of Carbapenem-resistant Enterobacterales (CRE) acquisition in inpatients with 3rd generation cephalosporin-resistant (3GCR) Enterobacterales at a tertiary-care hospital in Lebanon, and suggested a risk prediction score for it. This is a retrospective matched case–control study of inpatients with 3GCR Enterobacterales that are carbapenem resistant (cases) versus those with carbapenem-sensitive isolates (controls). Data analysis was performed on IBM SPSS program, version 23.0 (Armonk, NY, USA: IBM Corp.). Categorical variables were compared between cases and controls through bivariate analysis and those with statistical significance (*P* < 0.05) were included in the forward stepwise multiple logistic regression analysis. To develop the CRE acquisition risk score, variables that maintained statistical significance in the multivariate model were assigned a point value corresponding to the odds ratio (OR) divided by the smallest OR identified in the regression model, and the resulting quotient was multiplied by two and rounded to the nearest whole number. Summation of the points generated by the calculated risk factors resulted in a quantitative score that was assigned to each patient in the database. Predictive performance was determined by assessing discrimination and calibration. The sensitivity, specificity, positive predictive value, negative predictive value, and accuracy were calculated for different cutoffs of the score. The incidence of CRE acquisition significantly increased with time from 0.21 cases/1000 patient-days (PD) in 2015 to 1.89 cases/1000PD in 2019 (r^2^ = 0.789, *P* = 0.041). Multivariate analysis of matched data revealed that the history of cerebrovascular disease (OR 1.96; 95% CI 1.04–3.70; *P* = 0.039), hematopoietic cells transplantation (OR 7.75; 95% CI 1.52–39.36; *P* = 0.014), presence of a chronic wound (OR 3.38; 95% CI 1.73–6.50; *P* < 0.001), endoscopy done during the 3 months preceding the index hospitalization (OR 2.96; 95% CI 1.51–4.73; *P* = 0.01), nosocomial site of acquisition of the organism in question (OR 2.68; 95% CI 1.51–4.73; *P* = 0.001), and the prior use of meropenem within 3 months of CRE acquisition (OR 5.70; 95% CI 2.61–12.43; *P* < 0.001) were independent risk factors for CRE acquisition. A risk score ranging from 0 to 25 was developed based on these independent variables. At a cut-off of ≥ 5 points, the model exhibited a sensitivity, specificity, positive predictive value, negative predictive value, and accuracy of 64.5%, 85.8%, 82%, 70.7% and 75%, respectively. We also showed that only meropenem consumption intensity and CRE acquisition incidence density showed a strong positive correlation(r = 0.798, *P* = 0.106), unlike imipenem (r = − 0.868, *P* = 0.056) and ertapenem (r = 0.385, *P* = 0.522). Patients with a score of ≥ 5 points in our model were likely to acquire CRE. Only meropenem was associated with CRE carriage. Our proposed risk prediction score would help target surveillance screening for CRE amongst inpatients at the time of hospital admission and properly guide clinicians on using anti-CRE therapy.

## Introduction

With the turn of the century, the upsurge of antimicrobial resistance (AMR) has become a worldwide public health threat and its control has become a global priority^1^. The past 2 decades have brought a challenge to the clinical arena, as gram-negative organisms resistant to carbapenems are emerging and spreading rendering the latter increasingly ineffective. In Lebanon, reported 3rd generation cephalosporin resistance rates have reached 42% in *E. coli* and 37% in *Klebsiella* spp.^2,3^. Risk factors for 3rd generation cephalosporin resistance acquisition among Enterobacterales have been previously studied in our country^4–6^. Several investigators have shown that recent and recurrent antibiotic use, especially 3rd generation cephalosporins, recent surgery, use of invasive devices such as urinary catheters and mechanical ventilators, and multiple hospitalizations are independently associated with 3rd generation cephalosporin resistant Enterobacterales acquisition^4–6^. Consequently, in daily practice, clinicians have been acquainted with predicting 3rd generation cephalosporin resistance among patients infected with enterobacteriales, and this has been reflected in antibiotic choices proposed in Lebanese national therapeutic guidelines on the management of urinary tract infections, complicated intra-abdominal infections and febrile neutropenia in cancer patients^7–9^.

More recently, Carbapenem resistant Enterobacterales (CRE) are increasingly reported worldwide^10^. Lebanon is at risk for this alarming situation where an acute increase in prevalence has been seen in the country since identification of its first cases in 2008^2,3,11–13^. This problem is not only confined to the clinical setting in hospitals but is also being disseminated to the environment and community as well^2,3,11–15^. Infections caused by CRE pose a serious threat to an optimal inpatient care, as these organisms demonstrate resistance to many classes of antibiotics, thus limiting the available therapeutic options and leading to a poor clinical outcome, especially in resource-limited settings.

Predicting CRE carriage or acquisition is crucial to decide upon prompt and adequate empiric antibiotic therapy in infected patients and to implement infection control interventions to prevent the spread of these organisms in the hospital setting.

The aim of this study was to determine the progression with time of the incidence density of CRE carriage in inpatients with 3rd Generation Cephalosporin Resistant (3GCR) Enterobacterales at an acute tertiary care hospital in Lebanon over a period of 5 years and to identify independent risk factors associated with this carriage/infection, thus aiming to develop a risk prediction model for CRE acquisition. We studied the effect of the prior prescription of each carbapenem alone (imipenem, meropenem or ertapenem) on the risk of CRE acquisition, to check whether there is a difference among the members of the same class of carbapenems in induction and/or selection for CRE. We also studied the types of CRE species retrieved from clinical samples with their corresponding antibiogram. We also evaluated the types of CRE-related infections in our cohort, in addition to corresponding patient outcome. Moreover, we studied the correlation between CRE incidence density and the consumption intensity of imipenem, meropenem, and ertapenem to see if there is a difference between the different agents in causing resistance.

## Results

### Incidence density of CRE acquisition

**Figure 1:**
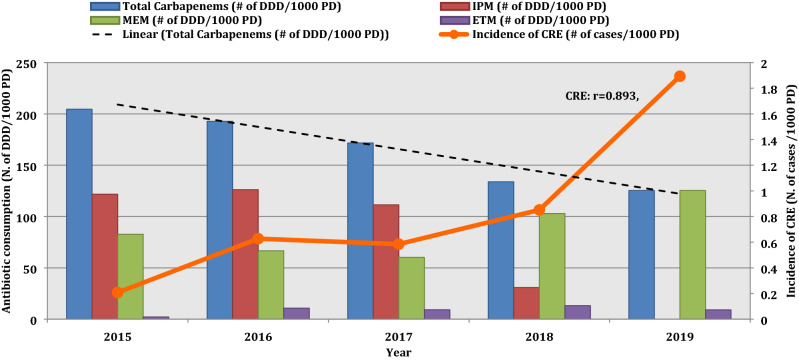
Temporal trend of carbapenem-resistant Enterobacterales acquisition incidence density and carbapenem consumption intensity from 2015 to 2019. *DDD* defined daily dose, *ETM* ertapenem, *IPM* imipenem, *MEM* meropenem.

Between January 2015 and December 2019, 1538 patients colonized and/or infected with 3GCR Enterobacterales were identified from the WHONET records of the hospital’s medical microbiology laboratory. Out of these patients, 155 cases acquired CRE and these were matched with 155 controls with carbapenem-sensitive 3GCR Enterobacterales. The incidence density of CRE acquisition was 0.21 cases/1000 PD and it significantly increased over the years showing an ascending trend reaching to 1.89 cases/1000 PD in 2019 (r = 0.893, r^2^ = 0.789, *P* = 0.041) (Fig. 1).

### Demographic data and clinical characteristics of patients who acquired CRE

The patients' demographic and clinical characteristics are displayed in Table 1. The median age of the patients who acquired CRE was 66 years (IQR 57–80), and 45.2% of the patients were men (70/155 patients). As for comorbidities, cardiovascular disease and diabetes mellitus were most commonly seen in 80/155 patients (51.6%) and 67/155 patients (43.2%), respectively, followed by malignancy and cerebrovascular disease seen in 47/155 patients (30.3%) and in 45/155 patients (29%), respectively. Twenty seven percent of the patients had a chronic wound including diabetic and pressure ulcers (47/155 patients). Within 3 months prior to CRE acquisition, 25% of the patients underwent a surgical procedure (39/155 patients) and 17.4% underwent endoscopy (27/155 patients) (Table 1).

**Table 1:** Bivariate analysis of general characteristics, therapeutic devices and procedures performed, and use of antibiotics potentially associated with carbapenem-resistant Enterobacterales acquisition.

Characteristics	Patients with CRE (cases) (N = 155, %)	Patients with 3GCR-CSE (controls) (N = 155, %)	Unadjusted odds ratio (95% confidence interval)	P-value
**Demographic**				
Age (years) [median, interquartile range (IQR)]	71 (57–80)	76 (66–82)	–	–
Gender (male)	70 (45.2)	73 (47.1)	0.925 (0.592–1.446)	0.732
**Nationality**				
Lebanese	149 (96.1)	148 (95.5)	0.993 (0.062–16.029)	0.996
Syrian	1 (0.6)	1 (0.6)	0.993 (0.062–16.029)	1
Iraqi	1 (0.6)	1 (0.6)	0.795 (0.209–3.018)	1
Others	4 (2.6)	5 (3.2)	0.795 (0.209–3.018)	0.736
**Comorbidities and underlying conditions**				
Cardiovascular disease	80 (51.6)	80 (51.6)	1.000 (0.641–1.561)	1
Respiratory disease	27 (17.4)	25 (16.1)	1.097 (0.604–1.991)	0.761
Liver disease	3 (1.9)	6 (3.9)	0.490 (0.120–1.996)	0.501
Diabetes	67 (43.2)	66 (42.6)	1.027 (0.655–1.610)	0.909
Cerebrovascular disease	45 (29)	25 (16.1)	2.127 (1.226–3.690)	0.007
Neurological disease	26 (16.8)	27 (17.4)	0.955 (0.529–1.726)	0.88
Presence of a chronic wound	42 (27.1)	23 (14.8)	2.133 (1.210–3.761)	0.008
Renal disease	43 (27.7)	42 (27.1)	1.033 (0.627–1.702)	0.899
Dialysis	17 (11)	3 (1.9)	6.242 (1.790–21.760)	0.001
Malignancy	47 (30.3)	34 (21.9)	1.549 (0.928–2.584)	0.093
Acute leukemia	14 (9)	5 (3.2)	2.979 (1.046–8.484)	0.033
Other hematologic disease	11 (7.1)	5 (3.2)	2.292 (0.777–6.759)	0.123
Non-hematologic disease/solid tumor	25 (16.1)	23 (14.8)	1.104 (0.596–2.043)	0.754
Hematopoietic cell transplantation (allogeneic)	12 (7.7)	2 (1.3)	6.420 (1.412–29.182)	0.006
Surgery during the past 3 months of index admission	39 (25.2)	39 (25.2)	1.000 (0.599–1.670)	1
Colonoscopy during the past 3 months of index admission	11 (7.1)	9 (5.8)	1.239 (0.499–3.080)	0.644
Endoscopy during the past 3 months of index admission	27 (17.4)	9 (5.8)	3.422 (1.552–7.546)	0.001
Previous hospital admission during the past 3 months of index admission	92 (59.4)	78 (50.3)	1.442 (0.920–2.259)	0.11
Previous intensive-care unit (ICU) admission during the past 3 months of index admission	39 (25.2)	7 (4.5)	7.108 (3.067–16.473)	< 0.0001
Urinary catheter (UC) use before acquisition during the index admission	75 (48.4)	36 (23.2)	3.099 (1.902–5.049)	< 0.0001
Duration of UC use before acquisition during the index admission (days) (median, IQR)	17 (4.25–30.75)	5.5 (1–11)	–	–
Mechanical ventilation (MV) before acquisition during the index admission	30 (19.4)	6 (3.9)	5.960 (2.403–14.780)	< 0.0001
Duration of MV before acquisition during the index admission (days) (median, IQR)	10 (6–38.5)	3.5 (1.5–4.75)	–	–
Central venous catheter (CVC) use before acquisition during the index admission	29 (18.7)	7 (4.5)	4.866 (2.061–11.487)	< 0.0001
Duration of CVC use before acquisition during the index admission (days) (median, IQR)	14 (8–28)	8 (4–10)	–	–
Stay in the ICU before acquisition during the index admission	44 (28.4%)	9 (5.8%)	6.43 (3.01–13.73)	< 0.0001
Length of stay (LOS) in the ICU before acquisition during the index admission (days) (median, IQR)	10.5 (3.75–27.5)	9 (5–11)	–	–
**LOS before acquisition during the index admission (days) (median IQR)**	7 (0–28)	0 (0–3)	–	–
< 4 days	60 (38.7%)	119 (76.8%)	0.19 (0.12–0.31)	< 0.0001
≥ 4 and < 10 days	25 (16.1%)	15 (9.7%)	1.80 (0.91–3.55)	0.09
≥ 10 and < 20 days	19 (12.3%)	16 (10.3%)	1.21 (0.60–2.46)	0.59
> 20 days	51 (32.9%)	5 (3.2%)	14.71 (5.68–38.11)	< 0.0001
< 10 days	85 (54.8%)	134 (86.5%)	0.19 (0.11–0.33)	< 0.0001
≥ 10 days	70 (45.2%)	21 (13.5%)	5.26 (3.01–9.18)	< 0.0001
**Cumulative broad-spectrum antibiotic use within 3 months before acquisition**				
Carbapenem use	74 (47.7)	25 (16.1)	4.751 (2.792–8.083)	< 0.0001
Duration of carbapenem use (days) (median, IQR)	8 (3.25–17.75)	7 (2–11)	–	–
Imipenem use	20 (12.9)	15 (9.7)	1.383 (0.680–2.812)	0.37
Duration of imipenem use (days) (median, IQR)	8 (3.75–12)	4 (1–10.5)		
Meropenem use	57 (36.8)	10 (6.5)	8.434 (4.108–17.312)	< 0.0001
Duration of meropenem use (days) (median, IQR)	8 (2–17)	6.5 (3.25–11.25)	–	–
Ertapenem use	6 (3.9)	1 (0.6)	6.201 (0.738–52.129)	0.121
Duration of ertapenem use (days) (median, IQR)	3,5 (2.25–4.75)	–	–	–
Piperacillin/Tazobactam use^a^	26 (16.8)	18 (11.6)	1.53 (0.80–2.93)	0.19
Duration of Piperacillin/Tazobactam use (days) (median, IQR)	7 (5–12.5)	6.5 (2–8.75)	–	–
3rd/4th generation cephalosporins use^a^	17 (11.0)	12 (7.7)	1.47 (0.68–3.19)	0.33
Duration of 3rd/4th generation cephalosporins use (median, IQR)	5 (3–11)	4 (3–4.5)	–	–
Ciprofloxacin use^a^	8 (5.2)	8 (5.2)	1.00 (0.37–2.74)	1
Duration of ciprofloxacin use (days) (median, IQR)	9 (7.75–9.75)	2.5 (1.75–4.75)	–	–
**Site of acquisition of the organism in question**				
Community-acquired	14 (9)	51 (32.9)	0.202 (0.106–0.385)	< 0.0001
Nosocomial	92 (59.4)	40 (25.8)	4.198 (2.593–6.797)	< 0.0001
Healthcare-associated	49 (31.6)	64 (41.3)	0.657 (0.413–1.047)	0.077
All-cause mortality	57 (36.8)	35 (22.6)	1.994 (1.212–3.282)	0.006

^a^The results shown are with no subsequent carbapenem prescription.

Regarding the site of acquisition of CRE, 59.4% of the cases were attributed to nosocomial setting (92/155 patients), with an overall median length of hospital stay reaching to 7 days prior to CRE detection (IQR 0–28) (Table 1).

As for broad-spectrum antibiotic exposure during the last 3 months before CRE acquisition, 16.8% of the CRE cases (26/155 patients) received piperacillin/tazobactam with no subsequent carbapenems with a median duration of 7 days (IQR 5–12.5) (Table 1). Almost 48% of the patients in the CRE group received carbapenems (74/155 patients) with a median duration of 8 days (IQR 3.25–17.75), majorly meropenem (36.8%, 57/155 patients) and to a lesser extent imipenem (12.9%, 20/155 patients) and ertapenem (3.9%, 6/155 patients).

### Types of CRE, isolation sites and antibiotic susceptibility profile

**Figure 2:**
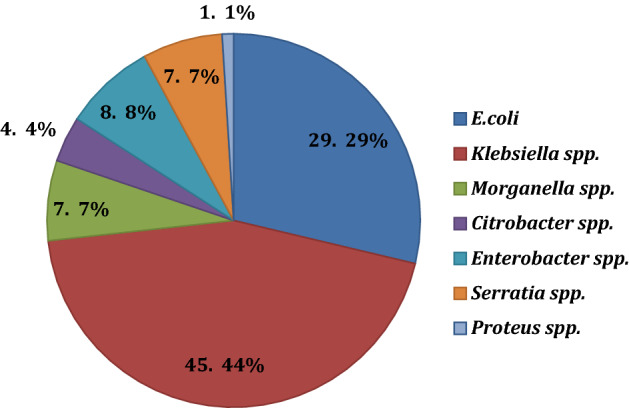
Types of carbapenem-resistant Enterobacterales species retrieved from clinical samples (%).

**Figure 3:**
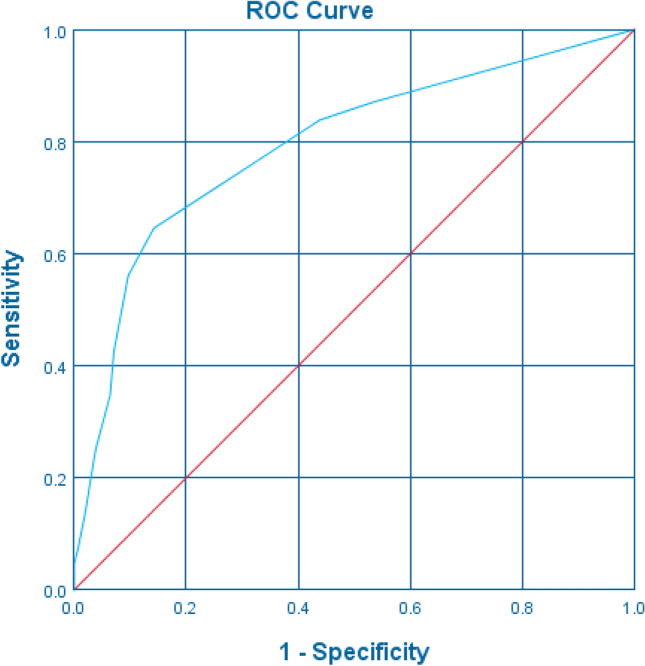
ROC curve for threshold selection of carbapenem-resistant Enterobacterales risk prediction score. Area under the curve = 0.793 [95% CI 0.742–0.844] (*P* < 0.001).

The types of CRE and distribution of isolation sites of the 155 isolates are shown in Figs. 2 and 3 (Table 2). The most common CRE pathogens were *Klebsiella* spp. (69/155 cases; 44.5%) followed by *E. coli* (29/155 cases; 29%). Other organisms included *Enterobacter* spp. (12/155 cases; 7.7%), *Morganella* spp. (11/155 cases; 7.1%), *Serratia* spp. (10/155 cases; 6.5%), *Citrobacter* spp. (6/155 cases; 3.9%), and *Proteus* spp. (2/155 cases; 1.3%) (Fig. 2). CRE were commonly isolated from urine (31%), wounds (26.5%), respiratory clinical specimens (sputum and DTA) (23.2%), blood (12.3%), stool samples/rectal swabs/perianal swabs (6.5%) and other body fluids (2.6%) (Table 2).

**Table 2:** Bivariate comparison between cases and controls regarding the type of isolated species and distribution of isolation site.

	Patients with CRE (cases) (N = 155, %)	Patients with 3GCR-CSE (controls) (N = 155, %)	Unadjusted odds ratio (95% confidence interval)	P-value
**Retrieved organisms**				
*E. coli*	45 (29.0%)	118 (76.1%)	0.13 (0.08–0.21)	< 0.0001
*Klebsiella* spp.	69 (44.5%)	21 (13.5%)	5.12 (2.93–8.95)	< 0.0001
*Morganella* spp.	11 (7.1%)	1 (0.6%)	11.76 (1.50–92.27)	0.003
*Citrobacter* spp.	6 (3.9%)	4 (2.6%)	1.52 (0.42–5.50)	0.52
*Enterobacter* spp.	12 (7.7%)	11 (7.1%)	1.10 (0.47–2.57)	0.83
*Serratia* spp*.*	10 (6.5%)	2 (1.3%)	5.28 (1.14–24.49)	0.02
*Proteus* spp*.*	2 (1.3%)	1 (0.6%)	2.01 (0.18–22.43)	0.56
**Clinical site of positive culture**				
Respiratory	36 (23.2)	15 (9.7)	2.82 (1.47–5.41)	0.001
Urine	48 (31)	88 (56.8)	0.34 (0.21–0.54)	< 0.0001
Wound	41 (26.5)	39 (25.2)	1.07 (0.64–1.78)	0.795
Blood	19 (12.3)	13 (8.4)	1.53 (0.81–1.94)	0.263
Other body fluids	4 (2.6)	3 (1.9)	1.34 (0.30–6.10)	1
Stool samples/rectal swabs/perianal swabs	10 (6.5)	0	NA	0.001
Others	2 (1.3)	3 (1.9)	0.66 (0.11–4.02)	1

As for differential susceptibility to carbapenems, 88.4% of the isolates were non-susceptible to imipenem, 35.5% to meropenem and 81.3% to ertapenem (Table 3). Around 27% of the isolates were resistant to one carbapenem only, 39% to 2 carbapenems and 34% to the 3 available carbapenems. Regarding other antibiotics, 81.3% of the isolated CRE were non-susceptible (intermediate or resistant) to 3GCs, 73.5% to ciprofloxacin, 63.9% to trimethoprim-sulfamethoxazole and 60.6% to amikacin. Susceptibility testing to tigecycline, colistin and to the new beta-lactam/beta-lactamase combinations (ceftolozane/tazobactam and ceftazidime/avibactam) was not performed on almost all of the isolated species (Table 3).

**Table 3:** Comparison of antibiotic susceptibility between different carbapenem-resistant Enterobacterales species.

Organism (N = 155, %)	IPM			MEM			ETP			3GC			AMK			SXT			C/T			CZA			COL			CIP		
	S	NS	NR	S	NS	NR	S	NS	NR	S	NS	NR	S	NS	NR	S	NS	NR	S	NS	NR	S	NS	NR	S	NS	NR	S	NS	NR
*E. coli* (n = 45, 29%)	2 (4.4)	39 (86.7)	4 (8.9)	2 (4.4)	9 (20)	34 (75.6)	1 (2.2)	40 (88.9)	4 (8.9)	1 (2.2)	39 (86.7)	5 (11.1)	33 (73.3)	7 (15.6)	5 (11.1)	9 (20)	31 (68.9)	5 (11.1)	0	1 (2.2)	44 (97.8)	0	0	45 (100)	5 (11.1)	0	40 (88.9)	5 (11.1)	35 (77.8)	5 (11.1)
Klebsiella (n = 69, 44.5%)	3 (4.3)	60 (87)	6 (8.7)	5 (7.2)	26 (37.7)	38 (55.1)	1 (1.4)	60 (87)	8 (11.6)	7 (10.1)	56 (81.2)	6 (8.7)	42 (60.9)	21 (30.4)	6 (8.7)	18 (26.1)	45 (65.2)	6 (8.7)	0	2 (2.9)	67 (97.1)	0	0	69 (100)	4 (5.8)	2 (2.9)	63 (91.3)	8 (11.6)	55 (79.7)	6 (8.7)
Morganella (n = 11, 7.1%)	0	11 (100)	0	7 (63.6)	2 (18.2)	2 (18.2)	9 (81.8)	2 (18.2)	0	6 (54.5)	5 (45.5)	0	9 (81.8)	2 (18.2)	0	6 (54.5)	5 (45.5)	0	0	0	11 (100)	0	0	11 (100)	0	0	11	7 (63.6)	3 (27.3)	1 (9.1)
Citrobacter (n = 6, 3.9%)	0	4 (66.7)	1 (16.7)	0	2 (33.3)	4 (66.7)	0	5 (83.3)	1 (16.7)	0	4 (66.7)	2 (33.3)	1 (16.7)	3 (50)	2 (33.3)	2 (33.3)	2 (33.3)	2 (33.3)	0	0	6 (100)	0	0	6 (100)	0	0	6 (100)	1 (16.7)	3 (50)	2 (33.3)
Enterobacter (n = 12, 7.7%)	0	11(91.7)	0	0	8 (66.7)	4 (33.3)	0	11 (91.7)	1(8.3)	0	11(91.7)	1(8.3)	5(41.7)	6 (50)	1(8.3)	2 (16.7)	9 (75)	1(8.3)	0	1 (8.3)	11 (91.7)	0	1 (8.3)	11 (91.7)	2 (16.7)	0	10 (83.3)	3 (25)	8 (66.7)	1 (8.3)
Serratia (n = 10, 6.5%)	0	10 (100)	0	0	6 (60)	4 (40)	0	7 (70)	3 (30)	1 (10)	9 (90)	0	2 (20)	8 (80)	0	5 (50)	5 (50)	0	0	1 (10)	9 (90)	0	0	10 (100)	0	0	10 (100)	2 (20)	8 (80)	0
Proteus (n = 2, 1.3%)	0	2 (100)	0	0	2 (100)	0	0	2 (100)	0	0	2 (100)	0	2 (100)	0	0	0	2 (100)	0	0	0	2 (100)	0	0	2 (100)	0	0	2 (100)	0	2 (100)	0
Total (N = 155, %)	5 (3.2)	137 (88.4)	11 (7.1)	14 (9)	55 (35.5)	86 (55.5)	11 (7.1)	126 (81.3)	18 (11.6)	15 (9.7)	126 (81.3)	14 (9)	94 (60.6)	47 (30.3)	14 (9)	42 (27.1)	99 (63.9)	14 (9)	0	5 (3.2)	150 (96.8)	0	1 (0.6)	154 (99.4)	11 (7.1)	2 (1.3)	142 (91.6)	26 (16.8)	114 (73.5)	15 (9.7)

*3GC* 3rd generation cephalosporins, *AMK* amikacin, *C/T* ceftolozane/tazobactam, *CIP* ciprofloxacin, *COL* colistin, *CZA* ceftazidime/avibactam, *ETP* ertapenem, *IPM* imipenem, *MEM* meropenem, *NR* not reported, *NS* non-susceptible, *S* susceptible, *SXT* sulfamethoxazole/trimethoprim.

### CRE-related infection types and patient outcome

Documented infections attributed to CRE were identified in 58.7% of the patients (91/155 cases), whereas 64 patients were colonized by CRE (41.3%) (Table 4). The most common types of infections were respiratory tract infections (27/155 cases; 17.4%), followed by wound and soft tissue infections (24/155 cases; 15.5%) bloodstream infections (22/155 cases; 14.2%), urinary tract infections (20 cases; 12.9%), and intra-abdominal infections (1 case; 0.6%). Clinical success was achieved in 44% of the patients with invasive CRE infections (40/91 cases), while microbiological success was documented in 33.9% (19/56 cases). The median length of hospital stay in patients after CRE acquisition was 12 days (IQR 9–15) compared to 8 days (IQR 6.5–9) for patients who acquired 3GCR-CSE in the control group (LogRank-test, P < 0.001). All-cause mortality in the group with infections reached 49.5% (45/91 cases) and that in the whole reached 36.8% (57/155 patients) (Table 4).

**Table 4:** Patient outcome and all-cause mortality stratified according to the type of infection caused by carbapenem-resistant Enterobacterales.

Type of CRE acquisition	Number of cases (N = 155, %)	Clinical outcome		Microbiological outcome			All-cause mortality (N = 155, %)
		Success (n, %)	Failure (n, %)	Success (n, %)	Failure (n, %)	Not determined (n)	
Colonization	64/155 (41.3%)	–	–	–	–	–	13/155 (8.4%)
Infection	91/155 (58.7%)	40/91 (44%)	51/91 (56%)	19/56 (33.9%)	37/56 (66.1%)	35	45/155 (29%)
Respiratory tract infections	27/155 (17.4%)	11/27 (40.7%)	16/27 (59.3%)	4/22 (18.2%)	18/22 (81.8%)	5	15/155 (9.7%)
Skin and soft tissue infections	24/155 (15.5%)	11/24 (45.8%)	13/24 (54.2%)	4/13 (30.8%)	9/13 (69.2%)	11	9/155 (5.8%)
Bacteremia	22/155 (14.2%)	5/22 (22.7%)	17/22 (77.3%)	8/15 (53.3%)	7/15 (46.7%)	7	17/155 (11%)
Urinary tract infections	20/155 (12.9%)	14/20 (70%)	6/20 (30%)	4/8 (50%)	4/8 (50%)	12	6/155 (3.9%)
Intra-abdominal infections	1/155 (0.6%)	0	1/1 (100%)	0	0	1	0

For each type of infection:

Clinical success (%) = (*Number of patients with clinical* success*/Total number of patients)* × 100.

*Clinical failure* (%) = (*Number of patients with clinical* failure*/Total number of patients)* × 100.

*Microbiological success* (%) = *Number of patients with microbiological* success/(*Total number of patients − Number of patients with undetermined microbiological response*)] × 100.

*Microbiological failure* (%) = *Number of patients with microbiological* failure/(*Total number of patients − Number of patients with undetermined microbiological response*)] × 100.

### Risk factors associated with CRE acquisition

The patients’ characteristics, comorbid conditions, hospital-based therapeutic interventions, use of invasive devices and history of previous antibiotic use were collected in the cases (CRE) and controls (3GCR-CSE) groups and compared through bivariate analysis (Table 1). Variables associated with CRE acquisition included: underlying cerebrovascular disease, acute leukemia, receipt of hematopoietic stem cells, intermittent hemodialysis, presence of a chronic wound, admission to the ICU during the during the 3 months preceding the index hospitalization, endoscopy during the 3 months preceding the index hospitalization, nosocomial site of acquisition of the organism in question, stay in the ICU during the same index admission, prior need for invasive procedures or devices during the index admission, including urinary catheters, mechanical ventilation and central-venous catheters, as well as the prior exposure to antibiotics within 3 months of CRE acquisition including carbapenems specifically meropenem, and piperacillin/tazaobactam for more than or equal to 10 days (Table 1).

Multivariate analysis for matched data showed that the history of cerebrovascular disease (OR 1.96; 95% CI 1.04–3.70; *P* = 0.039), history of hematopoietic cells transplantation (OR 7.75; 95% CI 1.52–39.36; *P* = 0.014), presence of a chronic wound (OR 3.38; 95% CI 1.73–6.50; *P* < 0.001), endoscopy done during the 3 months preceding the index hospitalization (OR 2.96; 95% CI 1.51–4.73; *P* = 0.01), nosocomial site of acquisition of the organism in question (OR 2.68; 95% CI 1.51–4.73; *P* = 0.001), and the prior use of meropenem within 3 months of CRE acquisition (OR 5.70; 95% CI 2.61–12.43; *P* < 0.001) were independent risk factors for CRE acquisition in inpatients (Table 5). Overall, the multivariate model displayed acceptable goodness of fit, correctly predicting CRE acquisition status in 75.5% of patients with a pseudo-R square (Nagelkere) of 0.356 (*P* < 0.001) and a Hosmer–Lemeshow goodness-of-fit statistic of 0.687.

**Table 5:** Independent risk factors associated with carbapenem-resistant Enterobacterales acquisition as determined multivariate logistic regression analysis and the assigned risk score points.

Variables	CRE vs. 3GCR CSE		
	Odds ratio (95% confidence interval)	P-value	Risk score point
History of cerebrovascular disease	1.96 (1.04–3.70)	0.039	2
History of hematopoietic cells transplantation	7.75 (1.52–39.36)	0.014	8
Presence of a comorbid chronic wound	3.38 (1.76–6.50)	< 0.001	3
History of endoscopy during the 3 months preceding the index hospitalization	2.96 (1.23–7.11)	0.015	3
Nosocomial acquisition of the organism in question	2.68 (1.51–4.73)	0.001	3
Prior use of meropenem within 3 months of acquisition	5.70 (2.61–12.43)	< 0.001	6

### CRE acquisition score

Based on odds ratios of the multiple logistic regression analysis, we created a potentially user-friendly tool to predict the probability of CRE acquisition using the following equation:

$$\begin{aligned} CRE \; acquisition \; score & = \left(History \; of \; cerebrovascular \; disease \times 2\right)+ \left(History \; of \; hematopoietic \;cells\; transplantation \times 8\right)\\ & \quad + \left(Presence \; of \; a \;comorbid \;chronic \;wound \times 3\right) + \left(History \; of \; endoscopy \; during \; the \; 3 \; months \; preceding \; the \; index \; hospitalization \times 3\right)\\ & \quad + \left(Nosocomial \; acquisition \; of \; the \; organism \; in \; question \times 3\right) + \left(Prior \; use \; of \; meropenem \; within \; 3 \; months \; of \; acquisition \times 6\right).\end{aligned}$$

In this formula, the presence of a variable was coded as 1, and its absence as 0. Accordingly, an individual risk score was generated for each patient, ranging from 0 to 25 (mean 4.36, 95% CI 3.9–4.8). ROC curve analysis suggested that the risk score could acceptably discriminate low risk vs. higher risk patients for CRE acquisition with an area under the curve of 0.793 (95% CI 0.742–0.844; *P* < 0.001) (Fig. 3). The performance characteristics of our score as a binary classification tool for CRE acquisition at selected cut-points is summarized in Table 6. Using a score cutoff 5 to indicate high risk of acquisition provided the best performance with a sensitivity, specificity, PPV, NPV and accuracy of 64.5%, 85.8%, 82%, 70.7% and 75%, respectively. The PLR and NLR associated with a ≥ 5 score cut-off were 4.55 and 0.41, respectively. When a score of 6 and above is used as a cutoff point to distinguish between potential CRE carriers and non-carriers, the sensitivity and specificity ratings were 56.1% and 90.3%, respectively. The PPV, NPV and accuracy were 85.3%, 67.3% and 73%, respectively. The PLR and NLR associated with a ≥ 5 score cut-off were 5.80 and 0.49, respectively (Table 6).

**Table 6:** Risk score performance characteristics for carbapenem-resistant Enterobacterales acquisition at different breakpoints

Score ≥	Proportion of patients N = 310(%)	TP (N)	FP (N)	TN (N)	FN (N)	Sensitivity (%)	Specificity (%)	PPV (%)	NPV (%)	PLR	NLR	Accuracy (%)
0	100	155	155	0	0	100	0	50	–	1.00	–	50
2	70.3	135	83	72	20	87.1	46.5	61.9	78.3	1.63	0.28	67
3	63.9	130	68	87	25	83.9	56.1	65.7	77.7	1.91	0.29	70
5^a^	39.4	100	22	133	55	64.5	85.8	82.0	70.7	4.55	0.41	75
6	32.9	87	15	140	68	56.1	90.3	85.3	67.3	5.80	0.49	73
8	24.8	66	11	144	89	42.6	92.9	85.7	61.8	6.0	0.62	68
9	20.6	54	10	145	101	34.8	93.5	84.4	58.9	5.40	0.70	64
11	14.5	39	6	149	116	25.2	96.1	86.7	56.2	6.50	0.78	61
12	7.7	21	3	152	134	13.5	98.1	87.5	53.1	7.0	0.88	56
14	3.9	11	1	154	144	7.1	99.4	91.7	51.7	11.0	0.94	53
15	2.3	7	0	155	148	4.5	100	100	51.2	–	0.96	52
17	1.6	5	0	155	150	3.2	100	100	50.8	–	0.97	52

*FN* False negative, *FP* false positive, *NLR* negative likelihood ratio, *NPV* negative predictive value, *PLR* positive likelihood ratio, *PPV* positive predictive value, *TN* true negative, *TP* true positive.

^a^Optimal breakpoint assigned using the Youden's J index.

### Correlation between CRE incidence density and carbapenem consumption

The temporal trends of CRE incidence density and the consumption of imipenem, meropenem, and ertapenem are shown in Fig. 1. Total imipenem consumption intensity strongly decreased over the years from 122 DDD/1000 PD in 2015 to zero in 2019 (r = − 0.917, r^2^ = 0.841, *P* = 0.06). Unlikely, meropenem consumption intensity showed an increase from 83 DDD/1000 PD in 2015 to 125 DDD/1000 PD in 2015, with a moderately increasing temporal trend (r = 0.717, r^2^ = 0.514, *P* = 0.172). Yet, ertapenem consumption slightly increased over the years from 2 DDD/1000 PD to 9 DDD/1000 PD (r = 0.625, r^2^ = 0.391, *P* = 0.259). On the other hand, total carbapenem consumption intensity significantly dropped from 204 DDD/1000 PD in 2015 to 125 DDD/1000 PD in 2019 (r = − 0.980, r^2^ = 0.961, *P* = 0.004). Meropenem consumption intensity and CRE acquisition incidence density showed a strong positive correlation (r = 0.798, r^2^ = 0.638, *P* = 0.106), unlike imipenem consumption intensity that demonstrated a strong negative correlation with it (r = − 0.868, r^2^ = 0.755, *P* = 0.056). On the other hand, ertapenem consumption was weakly positively correlated with CRE acquisition in this study (r = 0.385, r^2^ = 0.148, *P* = 0.522).

## Discussion

Carbapenem-resistant Gram-negative pathogens have progressively disseminated to different countries worldwide, presenting a serious public health concern^10^. CRE is increasingly reported in Lebanon and in the whole Middle East region^2,3,10,13^. A compilation of antibiotic susceptibility data of different pathogenic bacteria isolated from various types of clinical specimens from 13 Lebanese hospital laboratories during 2015 and 2016 showed that 40% of the isolated Enterobacteriaceae were resistant to 3GCs and that 3% were resistant to carbapenems^2^. In our study herein, the incidence of CRE carriage among inpatients significantly increased from 0.21 cases per 1000 PD in 2015 to 1.89 cases per 1000 PD in 2019 (r = 0.893, r^2^ = 0.789, *P* = 0.041). Our results are in line with previous findings reported in a retrospective study describing the temporal trends of antibiotic resistance of priority organisms among hospitalized patients at another tertiary care center in Lebanon^13^. Investigators reported a substantial increase in the incidence density of carbapenem-resistant *E. coli* from 0 per 10,000 PD in 2010 to 4.44 per 10,000 PD in 2018 (r = 0.91; *P* < 0.001) and in carbapenem-resistant K. pneumoniae from 0 per 10,000 PD in 2010 to 7.7 per 10,000 PD in 2018 (r = 0.54; *P* = 0.14)^13^.

Since 2008, heterogenous genetic contributors to multi-drug resistance among Enterobacterales especially to carbapenems were reported in several studies in Lebanon including carbapenemase production (predominantly OXA-48), acquired AmpC cephalosporinases, hyperproduction of extended-spectrum-β-lactamases, coupled with porin mutations or the overexpression of efflux pumps^15–17^. In our CRE cohort, high resistance rates to multiple antibiotic classes were recorded apart from carbapenems, where resistance to aminoglycosides, TMP/SMX and quinolones ranged between 60 and 74%, not to mention that more than 80% of the isolates were resistant to 3GCs. In a retrospective case series of CRE infections involving 40 patients set in a single hospital in Lebanon from 2011 to 2014, variable non-susceptibility to different antibiotics was also detected among the isolates^18^. Around 30% of the strains were resistant to amikacin and colistin, more than 75% were quinonlone-resistant; yet no resistance to tigecycline was detected in this cohort^18^.

Over the past two decades, the emergence and spread of CRE have been challenging treating physicians because related infections are difficult-to-treat and are associated with significant mortality, thus they present a clinical and economic burden in different healthcare settings worldwide^19–21^. In fact, our study demonstrated the clinical burden of CRE on the healthcare system, which was reflected through the low rates of clinical success in different types of infections (44%) like in pneumonia (40.7%) and bacteremia (22.7%). In addition, the median length of hospital stay in patients after CRE acquisition was 12 days (IQR 9–15) compared to 8 days (IQR 6.5–9) for patients who acquired 3GCR-CSE in the control group (LogRank-test, p < 0.001). All-cause in-hospital mortality in the group with CRE infections reached 49.5% (45/91 cases) and that in the whole reached 36.8% (57/155 patients). Our results echo those mentioned in the aforementioned case series in Lebanon, where 40% of the studied patient population had persistence or progression of their infection despite treatment and 58% of the patients had sepsis^18^. The in-hospital mortality rate in this series was 27.5%^18^.

The clinical outcome of patients with infections caused by 3GCR yet carbapenem-susceptible *Enterobacterales* in the control group is well established in the literature, especially with the use of carbapenems^22^. The latter are the gold standard therapeutic option for severe infections to which all newer antimicrobials are compared to for approval^23–25^. Carbapenems are widely available in Lebanon and are mentioned in almost all treatment guidelines whenever 3GCR *Enterobacterales* are suspected^7–9^.

However, the treatment of CRE in Lebanon was not standardized during the study period, and the recently approved beta-lactam/beta-lactamase combinations like ceftazidime/avibactam or the novel siderophore cephalosporin, cefiderocol were not available in the country. In 2020, the IDSA released new treatment guidelines for antibiotic resistant gram-negative infections with a special focus on CRE^26^. These guidelines recommended the use of new beta-lactam/beta-lactamase combinations in the management of CRE-related infections^26^. In resource-limited settings like Lebanon, even after the availability of these drugs in the market after regulatory approval, price consideration and other pharmacoeconomic factors play a major role in the unsustained availability of these new therapeutic options at patient bedside, thus the treatment outcome of CRE-related infections becomes a matter of uncertainty.

Our study was conducted to identify risk factors for harboring CRE in hospitalized patients and to develop a risk prediction model for CRE acquisition. Accordingly, knowing the risks of acquisition and trying to avoid them, if possible, would help mitigate the spread of these organisms and decrease the burden of associated infections on the health system and human lives. We identified five independent factors associated with CRE, namely being an HCT recipient, having a history of cerebrovascular diseases, having a chronic wound or ulceration, endoscopy done during the 3 months preceding the index hospitalization, the hospital setting as a source of acquiring the organism, and recent exposure to meropenem within 3 months of acquisition.

Risk factors for the carriage of CRE have been studied in many hospital-based cohort and cross-sectional studies that largely included patients who had already been hospitalized and were thus exposed to many medical interventions and antimicrobial treatment courses. Being an HCT recipient and having a chronic wound or ulcer, in addition to history of cerebrovascular disease, majorly including cerebrovascular accidents, were factors identified in other recent studies^27^. This may be explained by the recurring admissions and discharges as well as a relatively longer length of stay in this sick bedridden patient population prone to developing recurrent infections and exposing them to greater risk than the general patient population, not to mention the prolonged exposure to multiple courses of antibiotics^28,29^. It is worth noting that HCT is the only available type of transplantation being performed in our center where this fact might have created a sort of bias regarding which type of transplant could have been associated with CRE acquisition. In solid organ transplantation (SOT), CRE infections have been increasingly reported where in endemic settings its prevalence might reach up to 20% in this immunocompromised patient population^30^. SOT itself has also been independently associated with the development of CRE infection^31,32^. Exposure to healthcare facilities was also reported an independent risk factor for CRE infection^27,33^. Invasive procedures with scope devices were also identified as independent risk factors of CRE acquisition in matched case control studies^34,35^. The increased risk for CRE may arise owing to the inaccessibility to clean certain mechanical aspects of scope devices even when manufacturer standards are followed^35^. Cleaning and disinfecting scope devices is a hot issue where they pose a risk owing to the challenging nature of cleaning certain mechanisms and areas within them^35^.

Our study identified similar risk factors that other investigators highlighted such as exposure to broad-spectrum antibiotics including cephalosporins, carbapenems, fluoroquinolones and aminoglycosides^35–37^. On the other hand, our results also suggest that imipenem, meropenem and ertapenem have different powers in inducing or selecting for carbapenem resistance in Enterobacterales. A previous exposure to imipenem or ertapenem was not identified as a potential contributor to the acquisition of CRE based on the bivariate analysis, unlike meropenem that was identified as an independent risk factor increasing the odds of acquisition by almost 6 times, as per multivariate analysis. In addition, the increasing incidence density of CRE was strongly correlated with meropenem consumption, unlike imipenem and ertapenem consumption that showed a strong negative correlation and a slight positive correlation with it, respectively. In the same class of antibiotics, there are strong and weak inducers of resistance. For instance, ertapenem has been suggested as a treatment option when clinically appropriate in carbapenem-sparing stewardship strategies due to its weak ability in inducing carbapenem-resistance among Gram-negative bacilli^38,39^. Available data from several published studies in recent years showed that Ertapenem did not impact antipseudomonal carbapenem susceptibilities and in some it even demonstrated an improvement in carbapenem susceptibility among *Pseudomonas aeruginosa* and Enterobacterales^38,39^. Another potential explanation to the increasing incidence of CRE and its differential correlation with the consumption of each carbapenem alone might be attributed to horizontal gene transfer that allows these organisms to acquire and transmit genetic material from outside their clonal lineage. During the study priod, meropenem consumption intensity showed an increasing trend with time from 2015 to 2019. This increase has led to the emergence of carbapenem resistance among Enterobacterales, as shown previously, keeping in mind that the overall carbapenem consumption significantly dropped during the study period. The horizontal spread might have been attributed to the relatively heavier consumption of imipenem compared to meropenem between 2015 and 2017. In Lebanon, OXA-48 is the most prevalent carbapenemase in the country and the production of these enzymes is typically plasmid-mediated^15–17^. The transferable plasmids are known as potent contributors to the horizontal dissemination of carbapenem resistance genes, even when present in pathogens with MICs to carbapenems below the breakpoints for resistance^40^. This phenomenon emphasizes the importance of issuing antibiotic susceptibility results linked to detection of resistance genes and its association to adequate infection prevention and control measures.

On the other hand, these findings highlight the importance of implementing safe patient care practices to decrease the burden of acquisition as well as the significance of an antimicrobial stewardship program as a strategy for preventing CRE infections. Robust infection control measures should be taken proactively for patients with many risk factors to prevent CRE colonization or the transmission of CRE to other patients. Multifaceted interventions that help curb the spread of these pathogens in healthcare facilities include enhanced hand hygiene practice, minimizing device use, regular environmental cleaning and disinfection, and isolation through barrier/contact precautions and improve cohorting of patients or staff, not to mention active surveillance^41,42^.

Suggesting a score for CRE acquisition based on our independent risk factors through multivariate analysis would be helpful as an antibiotic stewardship tool. Technically, our scoring system demonstrated adequate calibration in our cohort and good discrimination, according to conventional thresholds to categorize score discrimination. From a clinical point of view, our score would guide prescribers on using empiric antimicrobial therapy that would take CRE into account in addition to other Gram-negative bacterial pathogens that might have overlapping risk factors like *Acinetobacter baumannii*^43^, *Pseudomonas aeruginosa*^44^, and *Stenotrophomonas maltophilia*^45^. On the other hand, there is no offhand rule for choosing cut-off values; we suggest using the patient’s functional status and clinical condition as a guide. For example, in a life-threatening infection with a high severity of illness score like sepsis where promptly initiating adequate empiric treatment is paramount, a score cut-off point with high sensitivity and a relatively acceptable specificity is chosen. In this case using a score cutoff 5 to indicate high risk of CRE acquisition provided the best performance with a sensitivity, specificity, PPV, NPV and accuracy of 64.5%, 85.8%, 82%, 70.7% and 75%, respectively. Empiric drug choice in an at-risk septic patient should take into account the roles and limitations of each therapy option. However, in a chronic condition where the patient is clinically stable and severity of illness scores are not high, we would choose a score cutoff 6 with a sensitivity, specificity, PPV, NPV and accuracy of 56.1%, 90.3%, 85.3%, 67.3% and 73%, respectively in order to guide the decision to treat empirically with anti CRE therapy pending confirmation by cultures, or when cultures could not be taken. The relatively high negative predictive value of our model at this cut-off point could spare the empiric anti-CRE coverage in potentially colonized patients or those at high risk of acquisition yet with a stable clinical status, which could cutback a significant overexposure to broad-spectrum antibiotics and selection of further resistant bacteria. The key is to stratify patients best suited to receive carbapenems in order to optimize patient outcomes whilst minimizing the potential for resistance selection and resultant collateral ecological damage.

Our study has some limitations. First, it is a single-center study with a limited sample size depending on the available cases. Second the laboratory diagnosis of CRE was phenotypic where resistance mechanisms and molecular typing were not available in our institution, given the importance of delineating the complexities of developed mechanisms of resistance and its effect on choosing an appropriate treatment. Third, our proposed score is institution specific where it is affected by the type of patients and their corresponding disease spectrum. For instance, among different types of cells and organ transplantation procedures, our data was skewed towards HCT, since it is the only type being performed at out facility. Our data does not analyze the effect of immunosuppression per se on the development of CRE; it rather describes an association between undergoing HCT and the acquisition of CRE. On the other hand, factors like duration of prior hospitalization or surgery during index hospitalization were not included among the clinical characteristics that were tested as risk factors of CRE acquisition. Another limitation lies in the fact that despite matching, the proportion of clinical sites is significantly different between cases and controls. Although this does not affect the variables included in the propensity score, these might have affected the estimation of this score. Nevertheless, our study, the first in Lebanon to our knowledge, emphasizes the need to identify risk factors of CRE acquisition in our population to effectively balance antimicrobial stewardship versus infection prevention and control measures in an area with increasing CRE endemicity. Furthermore, proposing a predictive score of acquisition to guide therapeutic decisions in CRE carriers based on the stratification of the patient’s clinical status may be helpful for avoiding unnecessary empiric antimicrobial therapy in low-risk patients, and for starting adequate treatment promptly in those at high risk.

## Conclusion

In Lebanon, CRE acquisition is increasing with time putting a high burden on the health care system. Antibiotic stewardship and robust infection control measures remain the major key tools for its mitigation. In our cohort of CRE carriers, the presence of chronic wounds and ulcers, history of endoscopy, history of HCT and cerebrovascular disease, the hospital as a clinical setting for acquisition, as well as broad-spectrum antibiotic use mainly meropenem were independent risk factors for this acquisition. Our results also suggest that imipenem, meropenem and ertapenem have different powers to induce or select for carbapenem resistance in Enterobacterales. A predictive model with these variables may be useful to identify patients at high versus low risk of carriage and avoid overuse of broad-spectrum therapy. However, further studies are needed to validate our results.

## Methods

### Setting and study design

Between January 2015 and December 2019, 1538 patients colonized and/or infected with 3GCR Enterobacterales were identified from the WHONET records of the hospital’s medical microbiology laboratory. Out of these patients, 155 cases acquired CRE and these were matched with 155 controls with carbapenem-susceptible 3GCR Enterobacterales.

This is a retrospective matched case–control study conducted on inpatients at Makassed General Hospital (MGH), a 186-bed university affiliated hospital and acute tertiary care referral center in Beirut from January 2015 to December 2019. Both cases and controls were identified and selected from the WHONET records of the hospital’s medical microbiology laboratory. Both were colonized and/or infected with 3GCR Enterobacterales. Cases were adults over 18 years of age, whom carbapenem-resistant 3GCR Enterobacterales were isolated from clinical cultures from any source during the defined study period. For each case, we randomly selected one control from adult inpatients admitted within the study period matched for age, gender, and date of hospitalization (within 30 days and within the closest time period to the case’s culture date). Patients in the control group acquired carbapenem-susceptible 3GCR Enterobacterales during their hospitalization. Cases with CRE isolated from multiple sites or on multiple dates were counted only once, where information from first event was collected as a case. For both cases and controls, organisms of the same species with the same antimicrobial susceptibility profile isolated from the same patient (matching hospital case number) were considered duplicate isolates and were removed from the analysis. The institutional review board (IRB) committee of Makassed General Hospital, Beirut, Lebanon, granted this study ethical approval. The IRB committee waived the requirement of informed consent from patients due to the retrospective nature of this study. During the data collection phase, only subject case numbers were included. At a later stage, a different number was assigned to each of our cases to safeguard subject privacy. All methods used were performed in accordance with the hospital’s IRB committee guidelines and regulations.

### Data collection

Demographic and clinical information were extracted from the patients’ electronic medical records and from hospital computerized databases according to a pre-prepared data collection sheet. Data analyzed for cases and controls included:

- Demographic characteristics including age, gender, and nationality.
- Comorbidities and underlying conditions including cardiovascular disease, respiratory disease, liver disease, cerebrovascular disease, neurologic disease, renal disease, diabetes mellitus, malignancy, hematopoietic cell transplantation (HCT), hemodialysis, and presence of pressure ulcers or chronic wounds.
- History of previous hospital admission and intensive care unit (ICU) admission during the past 3 months of index admission.
- Therapeutic devices and procedures performed including history of surgery during the past 3 months of index admission, history of gastro-intestinal procedures including colonoscopy and endoscopy during the past 3 months of index admission, mechanical ventilation, central venous catheterization, and urinary catheterization and their corresponding duration before acquisition during the same index admission.
- Stay in the ICU before acquisition during the same index admission and the length of hospital stay (LOS) before acquisition.
- All Enterobacterales retrieved with the type of clinical specimen and antibiogram, in addition to the site of acquisition of the organism in question [community-acquired (positive culture identified within 48 h of admission to the hospital, nosocomial (positive culture identified more than 48 h after hospital admission), or healthcare-associated (positive culture identified within 48 h of admission, yet the patient had been transferred directly from another healthcare facility or discharged within the 30 days preceding hospital admission].
- Broad-spectrum antibiotics prescribed before the positive culture in question with the corresponding duration of therapy including: carbapenems (imipenem, meropenem, ertapenem), piperacillin tazobactam, third and fourth generation cephalosporins, and ciprofloxacin. Antibiotic exposure was recorded till 3 months before acquiring the organism in question. When more than one of the aforementioned antibiotics was prescribed, we took in to consideration only the most recently prescribed agent the patient was exposed to before acquiring the organism in question.
- All-cause mortality during the same hospitalization.

For all time-dependent parameters, we only took into account the duration preceding the date of acquisition.

### Classification of CRE carriage and patient outcome

For the CRE cases, we differentiated whether the species retrieved caused an infection or it was a colonizer (including screening cultures). Infections with the organism in question were defined as any positive culture that was associated with either local or systemic signs or symptoms of infection as judged by the treating team, and based on the clinical diagnostic criteria established by the US Center for Disease Control and Prevention (CDC) and National Healthcare Safety Network (NHSN) criteria^46^. Patients with a blood or any other sterile source culture positive were directly defined to infection.

Active microbiological screening for patients’ colonization status and acquisition of carbapenem-resistant organisms was performed during the study period in the ICU only^47^. Specimens from the throat, axillae, urine, and perineal area were routinely cultured, in addition to sputum and/or skin lesion (aspirate/biopsy/swab) when applicable^47^. These cultures were secured upon ICU admission and on a weekly basis, as long as the patient was still there and whenever the clinical situation necessitated^47^.

Clinical outcome, microbiological outcome and all-cause mortality during the same hospital stay were studied in the CRE group. Clinical success was defined as an improvement in signs and symptoms of the primary infection caused by CRE managed with a course of antimicrobials based on available susceptibility testing reports. Persistence or deterioration of the initial infection symptoms/signs requiring a change of antibiotic therapy, and/or an infection-related death occurring later than 48 h after the start of therapy was considered a clinical failure. Microbiological success was defined as the eradication of the organism causing the primary infection in follow-up cultures after therapy. Persistent identification of the same organism 72–96 h after initiating therapy was considered a microbiological failure. The response was considered indeterminate when follow-up cultures were not available to verify eradication.

### Temporal trend of CRE and correlation with carbapenem consumption

The incidence density of CRE was calculated as the number of cases who acquired these organisms per 1000 patient-days (PD). The PD number was the number of patients present in any given location (e.g., hospital or ward) at a single time during a 24-h period^48^. The number of PD per year was obtained from the nursing head office. Data on individual carbapenems (imipenem, meropenem, and ertapenem) consumption in all adult patients admitted to our facility between 2015 and 2019 were retrospectively collected from the databases of the hospital pharmacy. Antibiotic consumption was defined as the number of Defined Daily Dose (DDD) and was normalized per 1000 patient-days. DDD is the assumed average maintenance dose per day for a drug used for its main indication in adults, according to the WHO ATC/DDD classification^49^.

### Microbiological identification and antibiotic susceptibility

The identification of bacteria from all types of cultures was performed according to standard microbiological procedures. All microbiological methods were consistent with the Clinical and Laboratory Standards Institute (CLSI) guidelines^50^. Antibiotic susceptibility was determined using disk diffusion and interpreted in accordance to the CLSI breakpoints of each corresponding year as per hospital’s clinical microbiology laboratory protocol^50^. Third generation cephalosporin resistance (3GCR) was defined as the nonsusceptibility of Enterobacterales to cefotaxime, ceftriaxone and/or ceftazidime as per CLSI guidelines. In the control group herein, 3GCR Enterobacterales were susceptible to cefoxitin and to imipenem. Susceptibility to carbapenems was considered when the zone diameter on disk diffusion testing was ≥ 22 mm for ertapenem and ≥ 23 mm for imipenem or meropenem. Carbapenem non-susceptibility was considered when zone diameter on disk diffusion testing was ≤ 21 mm for ertapenem or ≤ 22 mm for imipenem or meropenem, then confirmed by E-test method for imipenem, meropenem and ertapenem. A strain was carbapenem-resistant when the minimum inhibitory concentration of imipenem and meropenem was ≥ 4 mg/mL or ertapenem ≥ 2 mg/mL^50^. The average turnaround time for bacterial identification and antibiogram results was 3 working days. Molecular identification of carbapenem resistance genes and other rapid diagnostic tests that detect antimicrobial resistance were not available in the hospital laboratory at the time of the study.

### Statistical analysis

Data analysis was performed on IBM Statistical Package for the Social Sciences (SPSS) program for Windows, version 23.0 (Armonk, NY, USA: IBM Corp.). Descriptive statistics included the frequency (percentage) for categorical variables and median (interquartile range, IQR) for continuous variables. Variables were compared between cases and controls through bivariate analysis to assess any statistical significance using chi-square, fisher’s exact, and Mann Whitney tests as appropriate. All P-values were two tailed and *P* < 0.05 was considered statistically significant. Statistically significant factors from the bivariate model were included in the forward stepwise multiple logistic regression analysis. Adjusted odds ratio (OR) and 95% confidence interval (CI) were reported to indicate the impact and significance of each variable in the multivariate model. Statistical significance in the multivariable analysis was set at *P* < 0.05. To develop the CRE acquisition risk score, variables that maintained statistical significance in the multivariate regression model were assigned a point value corresponding to the OR divided by the smallest OR identified in the regression model, and the resulting quotient was multiplied by two and rounded to the nearest whole number. Summation of the points generated by the calculated risk factors resulted in a quantitative score that was assigned to each patient in the database. The scoring system performance was assessed by determining discrimination and calibration. A logit model for CRE acquisition risk prediction was constructed, and the area under the receiver operator characteristic (ROC) curve with a 95% CI was calculated to evaluate the validity of the model and to quantify its discriminative capacity in predicting acquisition. Calibration was assessed using the Nagelkerke’s R-square and Hosmer–Lemeshow goodness-of-fit tests. Sensitivity and specificity, positive predictive value (PPV), negative predictive value (NPV), positive likelihood ratio (PLR), negative likelihood ratio (NLR), and accuracy were calculated for different cutoff points of this score. An optimal breakpoint was assigned using the Youden's J index. Temporal trends of bacterial resistance rates and carbapenem consumption were analyzed independently with linear correlation by year. Pearson’s correlation coefficient was used to describe the relationship between individual carbapenem consumption and CRE incidence density using measures on a per-year basis. A *P* < 0.05 was considered statistically significant.

### Ethics approval and consent to participate

The institutional review board (IRB) committee of Makassed General Hospital, Beirut, Lebanon, granted this study ethical approval. The IRB committee waived the requirement of informed consent from patients due to the retrospective nature of this study. During the data collection phase, only subject case numbers were included. At a later stage, a different number was assigned to each of our cases to safeguard subject privacy. The contributing authors only performed data entry and analysis as well as the drafting of the manuscript.

## Data Availability

The data that support the findings of this study are available from Makassed General Hospital but restrictions apply to the availability of these data, which were used under license for the current study, and so are not publicly available. Data are however available from the authors upon reasonable request and with permission of Makassed General Hospital.
